# Adsorptive Stripping Voltammetric Determination of Amaranth and Tartrazine in Drinks and Gelatins Using a Screen-Printed Carbon Electrode

**DOI:** 10.3390/s17112665

**Published:** 2017-11-18

**Authors:** Yeny Perdomo, Verónica Arancibia, Olimpo García-Beltrán, Edgar Nagles

**Affiliations:** 1 Facultad de Ciencias Naturales y Matemáticas, Universidad de Ibagué, Carrera 22 Calle 67, Ibagué 730001, Colombia; yeny.perdomo@unibague.edu.co (Y.P.); jose.garcia@unibague.edu.co (O.G.-B.); 2 Facultad de Química, Pontificia Universidad Católica de Chile, Vicuña Mackenna 4860, Santiago 7820436, Chile

**Keywords:** amaranth, tartrazine, drinks, gelatins, screen-printed carbon electrode, adsorptive stripping voltammetry

## Abstract

A fast, sensitive, and selective method for the simultaneous determination of one pair of synthetic colorants commonly found mixed in food products, Amaranth (AM) and Tartrazine (TZ), based on their adsorption and oxidation on a screen-printed electrode (SPE) is presented. The variation of peak current with pH, supporting electrolyte, adsorption time, and adsorption potential were optimized using square wave adsorptive voltammetry. The optimal conditions were found to be: pH 3.2 (PBS), E_ads_ 0.00 V, and t_ads_ 30 s. Under these conditions, the AM and TZ signals were observed at 0.56 and 0.74 V, respectively. A linear response were found over the 0.15 to 1.20 µmol L^−1^ and 0.15 to 0.80 µmol L^−1^ concentrations, with detection limits (3σ/slope) of 26 and 70 nmol L^−1^ for AM and TZ, respectively. Reproducibility for 17.7 µmol L^–1^ AM and TZ solutions were 2.5 and 3.0% (*n =* 7), respectively, using three different electrodes. The method was validated by determining AM and TZ in spiked tap water and unflavored gelatin spiked with AM and TZ. Because a beverage containing both AM and TZ was not found, the method was applied to the determination of AM in a kola soft drink and TZ in an orange jelly and a soft drink powder.

## 1. Introduction

Amaranth (E123. AM) and tartrazine (E102. TZ) are synthetic water-soluble azo dyes that are widely used in drinks, cake mixes, ice-creams, cereals, candies, wines, soups, salad dressings, jams, chocolates, and coffee, as well as in a variety of drugs and cosmetics. Color provides the first impression of a food taste. AM is used extensively to give reddish or brownish color to drinks, syrups, and sweets, while TZ is a bright orange-yellow powder. Thus, synthetic dyes can replace natural colors due to their several advantages such as low cost, high stability to light, oxygen, pH, and low microbiological contamination. However, recent studies have revealed that many of these synthetic food colors may be harmful to people’s health. It has been reported that under anaerobic conditions, azo dyes can be reduced to form aromatic amines. For example, azoreductases from intestinal bacteria, some environmental microorganisms, and, to a lesser extent, mammals, catalyze the cleavage of the azo bond to produce aromatic amines which may be toxic, mutagenic, and carcinogenic to animals [1,2,3]. The World Health Organization (WHO) along with the Food and Agriculture Organization (FAO) have recommended an acceptable daily intake of AM to be between 0 and 0.5 mg kg^−1^, and that of TZ to be 0.75 mg kg^−1^ [4,5]. For this reason, selective and accurate methods are required for the determination of AM and TZ in food and beverage samples. Several methods have been proposed for the analysis of synthetic dyes: spectrometry, chromatography, capillary electrophoresis, electrochemistry, and so on, and each technique presents its own advantages and drawbacks. The UV-VIS method is widely used for the analysis of specific synthetic dyes in food and drink samples, but when two or more dyes are present, the overlapping of the peaks does not allow analysis with reliable values. In such cases, chemometric techniques become an indispensable tool to overcome these problems. Siddiquee et al. [4] reported a spectrophotometric method with a linear range between 1.0 × 10^−5^ and 5.0 × 10^−4^ mol L^−1^, and a detection limit (DL) of 1.13 × 10^−6^ mol L^−1^. Wu et al. [6] developed an HPLC-UV method for the determination of five dyes in drinks and candies, obtaining a DL of 6.4 ng mL^−1^ for AM. On the other hand, Ma et al. [7] used an HPLC–UV–MS technique and reported DLs of 8.6 and 5.4 ng for AM and TZ, respectively.

Adsorptive stripping voltammetry (AdSV) is a useful technique for determining organic compounds with oxidizable or reducible groups, such as azo dyes, since it combines excellent sensitivity, selectivity, accuracy, and precision with low instrumentation cost. The electrochemical reduction of azo dyes was studied extensively many years ago with mercury electrodes (polarography and voltammetry with handing mercury drop electrode HMDE, mercury film electrode HgFE and so on), but electrochemical oxidation and detection using unmodified and modified electrodes is still missing. Table 1 summarizes some of the published work with the determination of AM and TZ using different electrodes. However, with the exception of the study reported by Ni et al. [8] for the simultaneous determination of AM, TZ, Sunset yellow, and Ponceau 4R by adsorptive voltammetry using an HMDE, most other reports are made for the detection of the presence of AM or TZ, but not of the two simultaneously. On the other hand, screen-printed electrodes (SPE) have proven to be very cheap, compact, and versatile for different types of analytes [9,10].

One possible way of enhancing the adsorptive process and the sensitivity of the method is the use of surfactants, whose beneficial effects are unpredictable, as they tend to interfere by competitive adsorption. Surfactants may affect the speed of the electrochemical reactions, increase the reversibility of the system, and/or increase the oxidation or reduction current. Gomez et al. [11] reported that the presence of cetylpyridinium bromide (CPB) enhanced the selectivity of the simultaneous determination of tartrazine and Sunset yellow because the signals were separated from 70 to 150 mV, which was sufficient to determine each dye accurately. However, the peak reduction current of TZ decreased.

The aim of this work is the determination of AM and TZ using an unmodified screen-printed carbon electrode. To the best of our knowledge, the use of this electrode for the simultaneous determination of AM and TZ has not yet been reported.

## 2. Materials and Methods

### 2.1. Apparatus

Cyclic voltammograms (CV) and square wave voltammograms (SWV) were obtained using a DropSens µStat 400 potentiostat. The working electrode was a screen-printed 4-mm diameter carbon electrode (DRP-110) provided by DropSens (Oviedo-Spain). It had a built-in Ag reference electrode and an auxiliary carbon electrode. A magnetic stirring stick was used to homogenize the solution during the accumulation time. The pH measurements were made using a Lovibond SD 50 pH meter.

### 2.2. Chemicals and Reagents

Water used for sample preparation, reagent dilution, and rinsing purposes was obtained from Wasselab Purifier System (ASTM D1193). All chemicals (acetic acid, phosphoric acid, nitric acid, sodium hydroxide, methanol, etc.) were of analytical grade from Merck (Darmstadt, Germany). Amaranth (AM) and tartrazine (TZ) were obtained from Aldrich. Stock solutions containing 0.71 mmol L^−1^ of AM and TZ were prepared in water. Phosphate buffer solutions (PBS) were prepared from H_3_PO_4_, NaH_2_PO_4_, or NaHPO_4_ (Merck), and adjusted to the required pH with NaOH or HCl solutions. Acetate buffer solutions were prepared with acetic acid, adjusted to the required pH with NaOH.

### 2.3. Cyclic and Square Wave Voltammograms

Buffer solution (0.01 mol L^–1^), 250-μL aliquots of 0.708 mmol L^−1^ AM and/or TZ solution (17.7 µmol L^−1^), and deionized water up to a final volume of 10.0 mL were added to the voltammetric cell. An accumulation potential of 0.00 V (E_ads_) was applied for 30 s (t_ads_) with constant stirring at 1000 rpm. After an equilibrium time of 10 s, voltammograms were recorded, while the potential was scanned from 0.00 to 1.00 V using square wave modulation with 10 mV step amplitude, 10 mV pulse amplitude, and a frequency of 15 Hz. Each voltammogram was repeated three times. The calibration curves were obtained and linear regression and detection limits were calculated. Validation of the methodology was carried out in tap water spiked with AM and TZ. The proposed method was applied to the determination of AM in kola soft drink and TZ in an orange gelatin and a soft drink powder. In order to eliminate matrix effects, the standard addition method was used. The DL was calculated from DL(x) = by 3σx/y/b where σx/y is the random error in x and y, and b is the slope assuming that errors occur mainly in the y-direction. On the other hand, in the cyclic voltammograms the potential was scanned from 0.3 to 1.2 V at a scan rate of 50.0 mV s^−1^ without accumulation.

## 3. Results and Discussion

### 3.1. Electrochemical Behavior of AM and TZ on SPCE as a Function of pH

The electrochemical reduction of azo dyes in two steps was reported many years ago using HMDE [27]. In this reduction, the azo group is involved: R–N = N–R' + 2e^–^+ 2H^+^ = R–NH–NH–R' and R–NH–NH–R' + 2e^–^ + 2H^+^ = R–NH_2_ + R'–NH_2_. For this reason, the signals of different dyes often overlap. However, electrochemical oxidations usually correspond to substituent groups such as hydroxyl, and greater selectivity can be achieved. For AM and TZ it has been reported that electrochemical oxidation involves an electron and one proton and it corresponds to oxidation of naphthyl-sulfonate and pyrazole groups, respectively [19,21].

**Figure 1:**
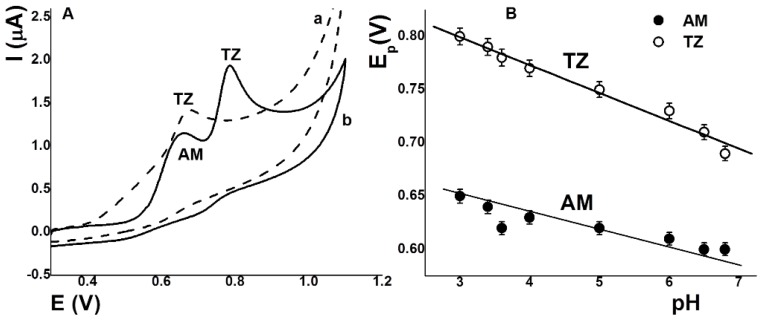
(**A**) Cyclic voltammograms (CV) of AM and TZ solutions (17.7 µmol L^–1^) in the presence of nitric acid solution (pH 3.2, curve a), and in the presence of phosphoric buffer solution (pH 6.8, curve b). Scan rate 50 V s^−1^. (**B**) Effect of pH on anodic peak potentials for AM (•) and TZ (o) using screen-printed carbon electrode.

The effect of the solution’s pH on the anodic stripping peak currents of AM and TZ using a screen-printed electrode was studied in the range from 3.2 to 6.8 with nitric acid and phosphate buffer solutions. Figure 1A shows the cyclic voltammograms (CV) for AM and TZ (17.7 µmol L^–1^) at pH 3.2 (curve a).

This figure shows two well-defined oxidation peaks at 0.66 V (ipa 0.37 µA) and 0.78 V (ipa 0.65 µA), which corresponded to the oxidation of AM and TZ, respectively. No reduction peak appears on the reverse scan, indicating the irreversible oxidation of these dyes. It was found that the peak potential shifted negatively when the pH increased (curve b), proving that protons are involved in the electrochemical reaction of AM and TZ. At pH 6.8, the signal of AM was only a slight inflection (0.54 V, ipa 0.01 µA) and the signal of TZ (0.68 V, ipa 0.56 µA) was extremely wide. Upon increasing the pH from 3.2 to 6.8, the currents decreased. pH 3.2 was used to achieve a sensitive and selective determination method. Plots with the relationship between the oxidation peak potentials of AM and TZ and pH are presented in Figure 1B. The slopes obtained for the E_pa_ vs. pH plot were 18 and 26 mV for AM and TZ, respectively. Wang et al. [12] reported values of 30 mV for AM using a carbon nanotube and polypyrrole composite-modified electrode. Meanwhile, Gan et al. [26] reported values of 55 mV for TZ using graphene decorated with nickel nanoparticles, and Yu et al. [17] reported values of 56 mV for TZ using an electrochemical sensor constructed with poly diallyldimethylammonium chloride-dispersed graphene and palladium nanoparticle composite. Both slopes of the above equations are close to the theoretical value of 59 mV/pH, suggesting an electrochemical process involving equal numbers of protons and electrons. Our values are more deviated from the theoretical values, which could be due to slow electrode reactions [19].

### 3.2. Effect of Supporting Electrolyte

**Figure 2:**
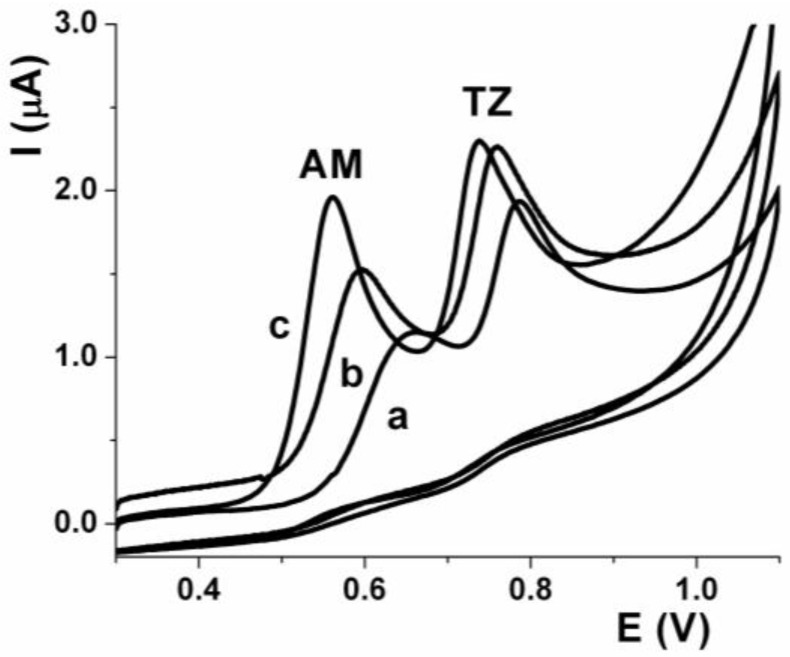
CV of AM and TZ (17.7 µmol L^–1^) solutions at pH 3.2; adjusted with nitric acid (curve a), acetate buffer (curve b), and phosphate buffer (curve c). Scan rate 50 V s^−1^.

The supporting electrolyte decreases the electrical resistance of the cell and reduces the Ohmic drop effect. On the other hand, it suppresses the migration of electro-active species towards the electrodes through electrostatic attractions to achieve diffusion-controlled currents. Figure 2 shows cyclic voltammograms of AM and TZ (17.7 µmol L^−1^, pH 3.2) recorded on SPCE in various solutions of supporting electrolytes with the addition of 300 µL of nitric acid (curve a), acetic buffer (curve b), or phosphoric buffer (curve c), all of them at a concentration of 0.01 mol L^−1^. For all the investigated solutions, two signals of well-defined equal oxidation peaks were seen in recorded CVs. With nitric acid the oxidation signals were found at 0.66 V and 0.79 V (0.36 and 0.78 µA), with acetic buffer the signals were found at 0.60 V and 0.76 V (0.69 and 0.95 µA), and with phosphoric buffer the signals were found at 0.56 V and 0.74 V (1.34 and 1.07 µA) for AM and TZ, respectively. On the other hand, the separation of the AM and TZ signals (∆E_pa_) were 0.13, 0.16, and 0.18 V for nitric acid, acetic buffer, and phosphoric buffer, respectively. According to these results, it can be concluded that the highest peak currents for AM and TZ (1.34 and 1.07 µA) and the greatest variation of ∆E_pa_ (about 180 mV) occurs with phosphoric buffer. All subsequent measures were carried out using phosphate buffer pH 3.2 (0.01 mol L^−1^), because it gave an adequate sensitivity and selectivity to determine AM and TZ simultaneously.

### 3.3. Influence of the Scan Rate (υ)

**Figure 3:**
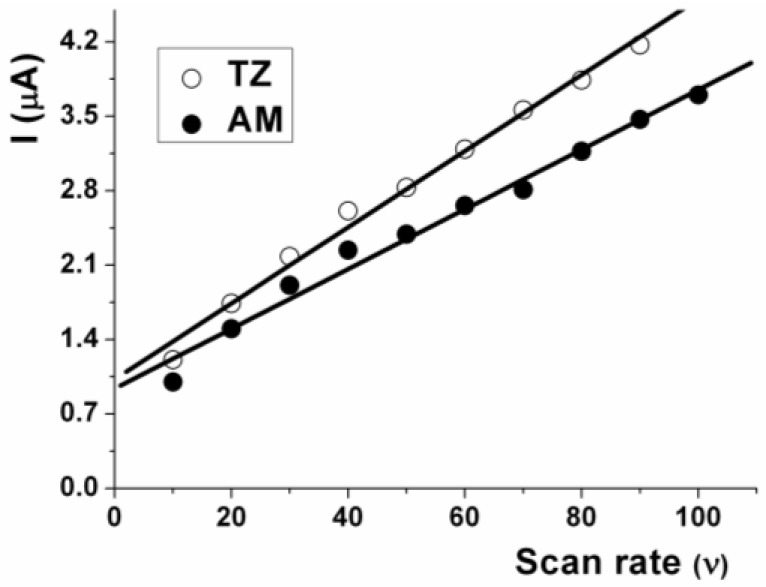
Effects of the scan rate on the anodic peak current of AM and TZ: Conditions: C_AM_ C_TZ_ 35.3 µmol L^–1^ at pH 3.2 (phosphate buffer 0.200.01 mol L^−1^).

With the purpose of identifying the process in the mass transport, the influence of the scan rate (v) on anodic peak currents for these azo dyes was studied using SPCE. Figure 3 show the plots with the relation of i_pa_ vs. scan rate (AM and TZ concentration 35.3 µmol L^−1^, pH 3.2). The oxidation peak currents of both dyes increased linearly with the scan rate in the range of 10–100 mV s^−1^ with the regression equations of i_pa_ = 1.071 + 0.034v (r = 0.994) for AM and i_pa_ = 0.919 + 0.028v (r = 0.992) for TZ. The linear relationship between the peak currents and scan rate suggested a predominantly adsorption-controlled process. Meanwhile, the oxidation peak potential (E_pa_) of AM and TZ was not shifted with the scan rate. Zhao et al. [9] reported that the oxidation peak current of AM increases linearly over the 20–400 mV s^−1^ range, while it also deviates from linearity from 400 to 1000 mV s^−1^, indicating the adsorption process changing to a diffusion process using a carbon nanotube and polypyrrole composite-modified electrode. Yu et al. [17] also assigned a mixed adsorption-diffusion driven oxidation process of AM using a Pd-doped polyelectrolyte functionalized graphene-modified electrode. On the other hand, Gan et al. [19] reported that the oxidation peak current of TZ increased linearly with the square root of the scan rate in the 100–400 mV s^−1^ range, indicating diffusion-controlled electrode processes, and the oxidation peak potential positively shifted with the scan rate using a graphene and mesoporous TiO_2_-modified carbon paste electrode.

### 3.4. Effect of Adsorption Potential and Time (E_ads_, t_ads_) on the Accumulation Step

The effect of the adsorption potential on the anodic peak currents for AM and TZ (5.0 µmol L^–1^) was studied in the range of −0.20 to 0.20 V using square wave stripping voltammetry. Anodic peak currents for AM and TZ increased when the potential was changed from −0.20 to 0.00 V, and at more positive values it decreased sharply. A potential accumulation of 0.00 V gives the highest anodic peak currents for AM and TZ, and it was chosen for further measurements. On the other hand, the effect of accumulation time was examined in the 0–60 s range. Peak current increased with increasing accumulation time prior to the potential scan, indicating that the AM and TZ are readily adsorbed on the SPCE. At first, the peak current of AM and TZ increased almost linearly with accumulation time until 30 s and then it tended to a steady value, probably due to electrode saturation. Results are not shown. On the basis of this result, 30 s was chosen for all measurements.

### 3.5. Effect of Instrumental Variables (Frequency, Step Potential, and Amplitude)

The square wave parameters studied were frequency, step amplitude, and pulse amplitude. Anodic peak currents of AM and TZ increased as all the parameters increased. However, when the frequency was higher than 10 Hz, the peaks of AM and TZ were very broad, losing resolution. A step amplitude of 10 mV and a pulse amplitude of 10 mV at a frequency of 10 Hz were used for further experiments.

### 3.6. Linear Range, Detection Limit, and Repeatability of the Method

**Figure 4:**
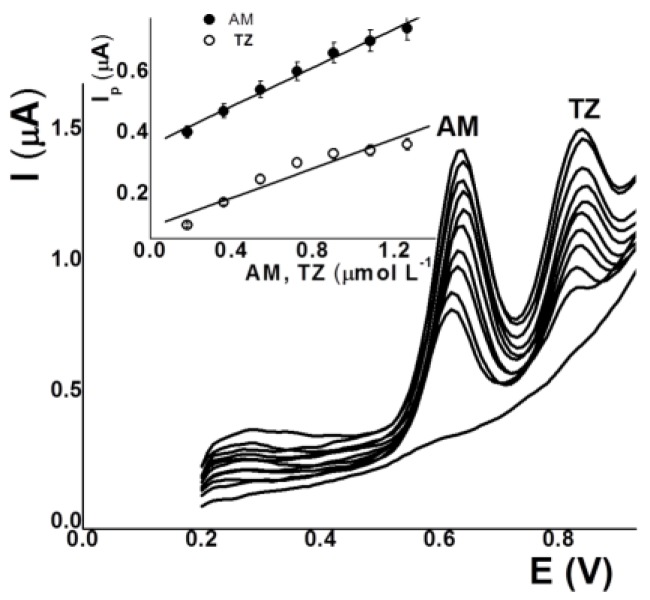
AdSV and calibration curves for increasing concentrations of AM and TZ with SPCE. Conditions: pH 3.2 (phosphate buffer); E_ads_ 0.00 V; t_ads_ 30 s.

Optimal analytical conditions were found to be: phosphate buffer pH 3.2 (0.01 mol L^−1^), E_ads_ 0.00 V, and t_ads_ 30 s. Under these conditions, the peak current was proportional to the concentration of AM over the 0.15–1.20 μmol L^–1^ range as well as proportional to the concentration of TZ over the 0.15–0.80 μmol L^–1^ range (Figure 4). The small linear range of TZ may be due to the simultaneous determination of these dyes and competitive adsorption. The DLs (3σ/b) obtained were 26 nmol L^−1^ for AM and 70 µmol L^−1^ for TZ. On the other hand, the repeatabilities were 2.5 and 3.0% (*n =* 7) for AM and TZ (17.7 µmol L^–1^), respectively, using three different electrodes.

### 3.7. Validation of the Method and Interference Studies

**Figure 5:**
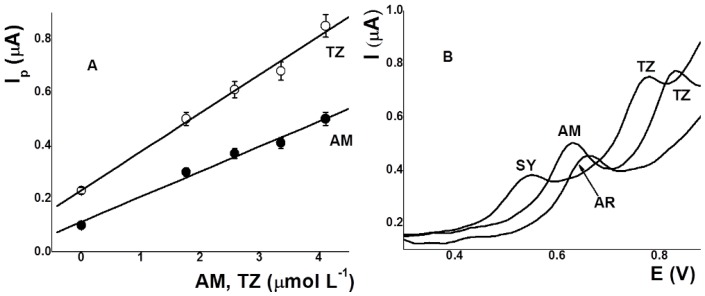
(**A**) Calibration curves of tap water spiked with AM and TZ (1.0 µmol L^−1^); (**B**) AdSV of different dyes: AM and TZ; SY and TZ; and AR (0.5 µmol L^−1^). Conditions: pH 3.2 (0.01 mol L^−1^ phosphate buffer), E_ads_ 0.00 V, t_ads_ 30 s.

Tap water spiked with AM and TZ was used for validation measurements. An aliquot of water was contaminated with these dye solutions (0.65 µmol L^–1^, sample 1) and the determination was carried out using the standard addition method, getting 0.80 ± 0.18 µmol L^–1^ for AM and 0.73 ± 0.12 µmol L^–1^ for TZ (Relative error, RE 23.0 and 12.3%, respectively). Due to these errors, more dye was added. A second aliquot of tap water (sample 2) was contaminated with 1.00 µmol L^–1^, obtaining 1.05 ± 0.10 µmol L^–1^ for AM and 1.13 ± 0.14 µmol L^–1^ for TZ (RE 5.0 and 13.0%, respectively). Figure 5A shows the plots obtained with sample 2. Moreover, the usefulness of the present method was validated using commercial unflavored gelatin spiked with AM and TZ. The values obtained were summarized in Table 2. The results showed that at lower concentrations of AM and TZ, the relative error was higher. On the other hand, these samples had complex compositions, with 12% protein.

In the voltammetric simultaneous determination of AM and TZ, the main interference may be caused by the presence of other dyes such as Sunset yellow (SY), Allura red (AR), Sudan I, Sudan II, and so on. Sudan I and Sudan II did not show signals under the conditions optimized for AM and TZ when they were added in concentrations 200 times greater than these. Instead, SY and AR were oxidized on a screen-printed carbon electrode. Square wave stripping voltammograms for AM, TZ, SY, and RA solutions (0.5 µmol L^−1^) are shown in Figure 5B. The experimental conditions were: pH 3.2 (phosphate buffer 0.01 mol L^−1^), Eads 0.00 V, and tads 30 s. The SWVs showed that SY, AM, RA, and TZ were oxidized at 0.55, 0.63, 0.67, and 0.78–0.83 V, respectively. These results indicate that only the simultaneous determination of SY-TZ (∆E: 230 mV), AM-TZ (∆E: 200 mV), and SY-RA (∆E: 120 mV) are possible. Interference in the determination of AM is produced by AR.

### 3.8. Application to Real Samples

**Figure 6:**
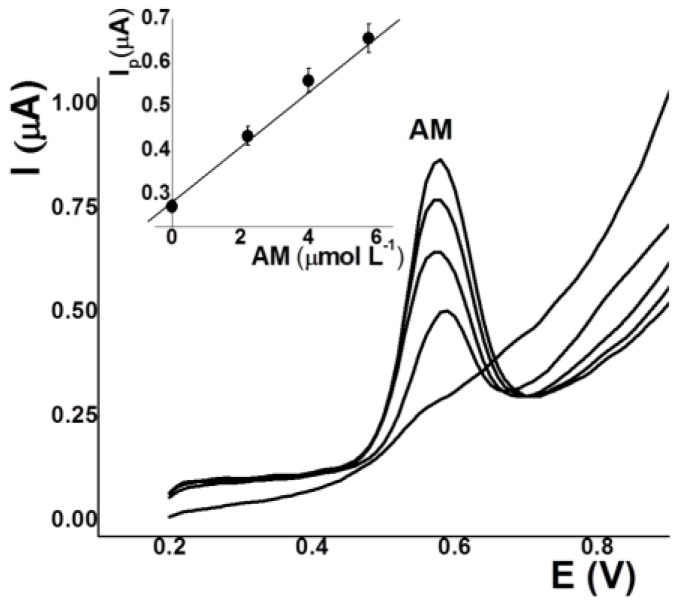
AdSV and calibration curves (inset) of kola soft drink for AM analysis. Conditions: pH 3.2 (0.01 mol L^−1^ phosphate buffer), E_ads_ 0.00 V, t_ads_ 30 s.

In order to apply the optimized methodology, we looked for drinks that contained both AM and TZ. However, this was not possible, as kola soft drinks contain only AM. Food manufacturers indicate what kind of dye or of dyes are present, but the amount is not specified. In addition, the ingredient information for kola soft drinks reported the use of AM. Using the standard addition method, three samples of kola soft drinks were analyzed obtaining 35.5 ± 0.50 µmol L^–1^ of AM (21.5 mg L^−1^). Figure 6 shows the square wave stripping voltammograms and calibration curve obtained for AM in a kola soft drink. On the other hand, to apply the methodology for the determination of TZ, orange gelatin and orange soft drink samples were analyzed, obtaining 10.6 ± 1.5 and 60.5 ± 2.5 µmol L^–1^ (5.7 and 32.3 mg L^−1^), respectively. These last two samples also contained 5.0 ± 1.0 and 10.0 ± 2.0 µmol L^–1^ (2.3 and 4.6 mg L^−1^) of SY. WHO and FAO recommend an acceptable daily intake between 0 to 0.5 mg kg^–1^ for AM, and of 0.75 mg kg^−1^ for TZ. Therefore, child weighing 30 kg should not consume more than 700 mL of kola and orange soft drinks daily.

## 4. Conclusions

A screen-printed carbon electrode was very adequate for the quick determination of AM in kola soft drinks and of TZ in gelatins and soft drinks. The calibration curve showed good linearity with only 30 s of accumulation. The DLs obtained for AM and TZ (15.7 and 37.4 µg L^−1^, respectively) were below the permitted limits of AM and TZ in commercial beverages (100 mg L^−1^). The same electrode without treatment was used for a series of measurements. The relative error of the validation was considerable at lower concentrations of 10.0 µmol L^−1^.

## Figures and Tables

**Table 1:** Electroanalytical methods for AM and/or TZ.

Dye	Electrode	Method	Recovery (%)	DL (µmol L^−1^)	Samples	Ref.
Amaranth Tartrazine	HMDE	reduction		--- ---	Foods	[8]
Tartrazine	HMDE	reduction		3.30 µg/L	Flavored gelatin	[11]
Amaranth	CNT–ppy-GCE	oxidation	93.0	0.0005	Fruit drinks	[12]
Amaranth	EGPE	oxidation	98.0	0.036	Grape juice	[13]
Amaranth	CNT/GO-IL-GCE	oxidation	95.0–105.0	0.0001	Foods	[14]
Amaranth	SPCE	oxidation		0.018	Soft drinks	[15]
Amaranth	HMDE	reduction	104.0	0.0017	Soft drinks	[16]
Amaranth	PDDA-Gr-Pd/GCE	oxidation		0.005	Soft drinks	[17]
Tartrazine	nAu-CPE	oxidation	96.0–104.0	0.002	Soft drinks	[18]
Tartrazine	GN–PTA-GCE	oxidation	95.0–104.0	30 µg/L	Soft drinks	[19]
Tartrazine	BDDE	oxidation	95.0	0.0627	Foods	[20]
Tartrazine	GN/TiO_2_-CPE	oxidation	99.0–102.0	0.008	Foods	[21]
Tartrazine	ERGO-SPCE	oxidation		0.0045	Foods	[22]
Tartrazine	HMDE	reduction		0.03	Soft drinks	[23]
Tartrazine	GCE	reduction		0.011 mg/L	Foods	[24]
Tartrazine	MIP-PmDB/PoPD-GCE	oxidation		0.0035	Soft drinks	[25]
Tartrazine	GN-Ni/GCE	oxidation		0.00108	Foods	[26]

GCE: Glassy carbon electrode. PSS-Gr-Pd/GCE: Pd-doped polyelectrolyte functionalized graphene modified electrode. nAu-CPE: Gold nanoparticle-modified. GN–PTA-GCE: Electropolymerized film of graphene layer-wrapped phosphotungstic acid-modified. BDDE: boron-doped diamond electrode. GN/TiO_2_-CPE: Graphene and mesoporous TiO_2_-modified. ERGO-SPCE: Reduced graphene oxide-modified screen-printed carbon electrode. CNT–ppy-GCE: Carbon nanotube and polypyrrole composite modified GCE. EGPE: Expanded graphite paste electrode. GO/CNT-IL/GCE: Graphene oxide, carbon nanotubes and ionic liquid modified GCE. MIP-PmDB/PoPD-GCE: Molecularly imprinted with dihydroxybenzene and o-phenylenediamine as monomers modified GCE. GN-Ni/GCE: Ultrathin graphene with nickel nanoparticles modified GCE electrode. MWCNT/PGE: Multi-walled carbon nanotube-modified pyrolytic graphite electrode.

**Table 2:** Determination of AM and TZ in commercial unflavored gelatin.

Unflavored Gelatin	Added (µmol L^−1^)		Found (µmol L^−1^)		% R. Error	
samples	AM	TZ	AM	TZ	AM	TZ
1	3.13	7.0	2.53	8.08	−19.0	15.4
2	1.61	1.68	2.00	1.31	24.2	−22.2
